# Evaluation of Acute and Subchronic Toxicity Induced by the Crude Ethanol Extract of *Plukenetia volubilis* Linneo Leaves in Swiss Albino Mice

**DOI:** 10.1155/2021/6524658

**Published:** 2021-10-19

**Authors:** Phuong-Nhung Thi Tran, Thi Thanh Nha Tran

**Affiliations:** ^1^Institute of Biotechnology and Food Technology, Industrial University of Ho Chi Minh City, 12 Nguyen Van Bao street, Go Vap District, Ho Chi Minh City, Vietnam; ^2^Faculty of Chemical Engineering, Industrial University of Ho Chi Minh City, 12 Nguyen Van Bao street, Go Vap District, Ho Chi Minh City, Vietnam

## Abstract

*Plukenetia volubilis Linneo* (*P. volubilis*), or *Sacha inca*, is an oleaginous plant from the Euphorbiaceae family. The presence of terpenoids, saponins, tannins, glycosides, phytosterols, and phenolic compounds in the ethanol extracts of *P. volubilis L* leaves has been reported, showing a range of bioactivities including antimicrobial, anti-inflammatory, antioxidant, and analgesia. However, the safety of this plant has not yet been reported explicitly. This study thus is aimed at evaluating the toxicity of the ethanol extract of *P. volubilis* leaves (EtPV) by acute and subchronic toxicity tests in Swiss albino mice following standard procedures set by The Organization for Economic Cooperation and Development (OECD) with slight modifications. In the acute toxicity test, the treatment groups were administered orally with the EtPV at doses of 1000, 3000, 5000, and 7000 mg/kg body weight in small fractions during 16 hours, and the mice were then observed in 14 consecutive days. In the subchronic toxicity study, the EtPV was given at doses of 100, 300, 500, and 700 mg/kg body weight for 90 days. Changes in behavior, mortality rate, and body and the weights of vital organs, hematology, clinical biochemistry, urine analysis, and histologic morphology were evaluated. The acute toxicity study showed that the EtPV causes no sign of toxicity or mortality. The hematological, biochemical and urine analyses, changes in the weight of the body and vital organs (heart, liver and kidney), and histopathological analyses of organs indicated no evidence of toxicity at any doses. It was also revealed that oral administration of EtPV is safe at the oral doses set by acute and subchronic toxicity tests, and the oral lethal dose for the EtPV is higher than 7000 mg/kg. This study is the first to confirm the safety of *P. volubilis* leaf ethanol extract, and as a result, encouraging further investigation to examine EtPV potential for traditional medicine.

## 1. Introduction

Historically, traditional medicine has been used in many countries to treat a wide range of diseases [1]. Isolated chemicals and bioactive compounds from plant materials have increasingly been reported as the main source of pharmaceutical drugs, which are either naturally derived or synthetic analogues of naturally occurring compounds. However, there is also evidence that some herbal extracts produced adverse effects during treatment process [2], arousing the concern about the safety of herbal medicine [3]. Consequently, the safety of plant materials needs to be examine before they can be used to manage ailments [4]. The importance of scientific investigation into the toxicity of indigenous herbal medicines has also been emphasized by WHO as part of the safety assessment of herbal products [5].

*Plukenetia volubilis L.*, commonly known as sacha inchi, is a perennial plant in the Euphorbiaceae family. It is native in tropical South America, some of the Windward Islands in the Caribbean, and cultivated commercially in South East Asia. The oil derived from the sacha inchi nut has been reported to contain a variety of unsaturated fatty acids, *γ*-, and *δ*-tocopherols, and traditionally used by indigenous people for cosmetic purposes, treating rheumatic problems and aching muscles for centuries [6].

More medicinal applications of the seeds and the oil of this plant have been discussed recently. The pharmacological value of the extract from *P. volubilis* plant was first noted in the reference [7]. Huamán et al. (2008) and Garmendia et al. (2011) used extracts of the seeds and leaves of *P. volubilis* to reduce the lipid profile of patients with postprandial lipemia [8] and lower cholesterol in patients with high blood cholesterol [9]. Another investigation from Ana Karina et al. (2013) on four extracts of fresh leaves *P. volubilis* including methanol (MEL), ethanol (EEL), chloroform (CEL), and aqueous (AEL) showed that they had antioxidant and antiproliferative activities against HeLa cells. The extracts were also able to stimulate cell proliferation in fibroblast cells-3T3 [10]. The use of *P. volubilis* as traditional medicine for cholesterol regulation, digestion improvement, and preventing diabetes or cognitive disorders has also been investigated [11].

In Vietnam, the extract of *P. volubilis* leaves (EtPV) has been consumed traditionally to prevent rheumatoid arthritis (RA), an autoimmune disorder of unknown etiology. This disease affects approximately 1% of the adult population globally with the occurrence depending on the genetic and environmental factors [12]. An investigation into the prevalence of RA in Vietnamese suggested that 0.3% of the urban Vietnamese population suffers from rheumatoid arthritis [13]. The standard treatment of this condition commonly begins with disease-modifying antirheumatic drug (DMARD) therapy within 3 months of diagnosis. However, the DMARDs only control but cannot cure the disease and therefore, prevention of this condition is highly recommended [14, 15]. Since methods for accurately predicting those at high risk of RA are still not available [14], herbal medicine such as the EtPV could be used as an alternative intervene to at least delay or may be completely inhibit the condition. However, the safety of plant extract has to be confirmed to avoid any health risks, and to the best of our knowledge, toxicological evaluation of ethanol extract of EtPV has not yet been conducted by any research group. This work, thus, is aimed at examining the acute and subchronic toxicity of the EtPV in Swiss albino mice, forming the foundation for subsequent investigations into the pharmacological effects of the EtPV in vivo.

## 2. Materials and Methods

### 2.1. Collection and Extraction of *P. volubilis* Leaves

**Figure 1:**
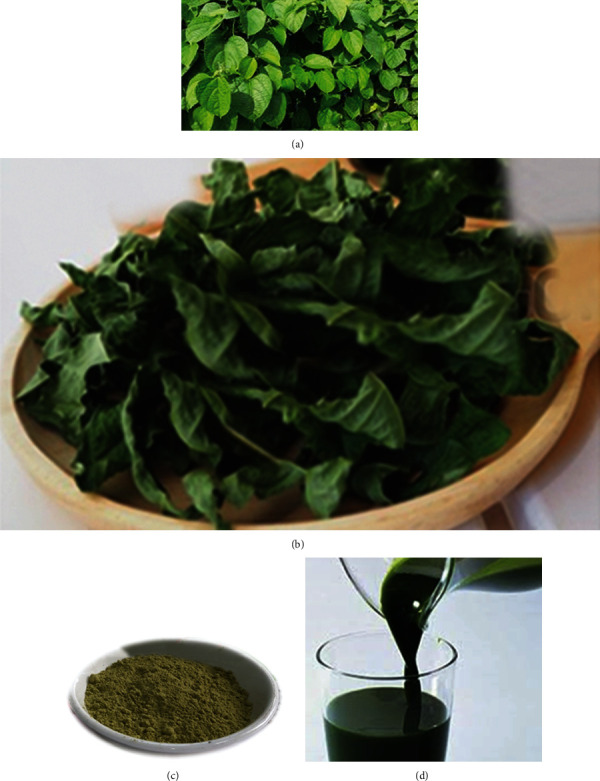
The collection of the extract of P. volubilis leaves. (a). The fresh form of P. volubilis leaves. (b) The dry form of P. volubilis leaves. (c) The dry powder of P. volubilis leaves. (d) The crude ethanol extract of P. volubilis leaves (EtPV).

*Plukenetia volubilis L*. leaves were collected from a farm named the Eastern Agricultural Company, located in Buon Ma Thuot city (12046′-12055′N, 10802′-108023′E), Daklak Province, Vietnam, in October 2019 (Figure 1(a)). No permission was required to collect the plant, and the collection of the plant did not cause risk of extinction for the species to any extent.

The plant sample was authenticated by a botanist at the Institute of Biotechnology and Food Technology, Industrial University of Ho Chi Minh City, Vietnam. The voucher specimen (sample code PV241019VST) was deposited in Department of Plant Biotechnology of the Institute of Biotechnology and Food Technology, Industrial University of Ho Chi Minh City, Vietnam. The leaves were washed and dried at 60°C to obtain samples with the moisture content less than 12% (Figure 1(b)). The process continued with the dried material being ground in a blender to powder and size uniformity was achieved by sieving the powder through a stainless-steel mesh with the diameter of 200 mm (Figure 1(c)). The leaf powder was then vacuum-packed in PE bags and stored at 0-4°C for subsequent experiments.

The extraction was carried out in 60% ethanol using the ultrasonic-assisted extraction method. One gram of sample was mixed with 20 ml solvent, placed in a sonication microwave (Sanyo, Japan) for 3 hours at a temperature window of 30-40°C. The extract was then filtered through the filter paper Whatman No. 4. The whole process was repeated twice with fresh solvent to obtain the crude extract (Figure 1(D)). The ethanol solution was evaporated and concentrated to achieve the EtPV which was kept frozen until being used [16].

### 2.2. Phytochemical Screening of Extract

Preliminary qualitative phytochemical analysis was carried out to identify the secondary metabolites in the ethanol extract of the *P. volubilis* leaves including phenolic compounds test: magnesium and HCl test [17], alkaloid test: Mayer test [18], saponin test: Froth test [19], flavonoid test: alkaline test [20], phytosterol test: Libermann-Burchard test [21], tannine test: iron chloride test [22], diterpene test: copper acetate test [22], sterol and triterpenoid test: Salkowski test [23], glycoside test: Borntrager test [24], carbohydrate test: Molishs test [21], protein and amino acid test: Biuret test [21], and oils and fats: saponification test [25].

### 2.3. Experimental Animals

Healthy Swiss albino mice male weight of 23-25 g, 6-week-old, were obtained from Pasteur Institute, Ho Chi Minh City, Vietnam. The animals were acclimated to laboratory conditions for 15 days. They were housed in standard glass cages and kept under the standard condition at a temperature of 23°C (±2°C), with a 12-hour light/12 hrs dark cycle [26]. In each assay model, 30 male mice were randomly assigned into five groups, one control group and four treatment groups [27] and captured in five different cages. Individuals were identified by marking on their tails using a permanent marker. The cage was made of sanitized glass with the length × width × height being 60 × 30 × 30 cm. The woodchip material used for cage lining was sprayed with Effective microorganisms (EM) to disinfect and remove odor. During the experiment, the cage lining was changed regularly (every 2-3 days). Mice were given ad libitum access to food and water [28]. The tests were performed following the Declaration of Helsinki strictly (2015) [29]. All animals received human care according to the Vietnam legislation on the usage and care of laboratory animals and according to of Ministry of Health Vietnam Guideline on ethics in biomedical research [30]. The study was approved by the Ethics committee of the Council for Scientific Research and Technology Development of the Ho Chi Minh City University of Industry, Vietnam (No. 98/HD-DHCN).

### 2.4. Acute Toxicity Study

The acute toxicity study of the ethanol extract of *Plukenetia volubilis* L. leaves was carried out using the “Up-and-Down” method of testing in Swiss albino mice set by the Organization for Economic Development (OECD) guideline No. 425 [31] with slight modifications. The modifications were based on Guidelines for preclinical and clinical trials of oriental medicine and herbal medicine from the Decision of Department of Science, Technology, and Training, Ministry of Health, Vietnam [32]. The dose was calculated based on the dry-matter content in the extract (1000, 3000, 5000, and 7000 mg/kg weight body) [33, 34].

The mice were randomly assigned into 5 groups (6 mice per group), one control group and four EtPV-treatment groups. The treatment groups were orally administered with the EtPV in 8 small fractions during 16 hours at doses of 1000, 3000, 5000, and 7000 mg/kg after fasting overnight, whereas the control group was given distilled water. General changes (changes in the skin, hair, eyes, mucous membranes, and respiratory, circulatory, autonomic, and central nervous systems, motor activity, convulsion, tremors, salivation, diarrhea, lethargy, or sleep) and signs of toxicity and mortality were observed for every hour during 24 h after dosing, and further observation was conducted for 14 consecutive days [35]. The animals were finally lethal by inhalation of CO_2_ on sacrifice day [36, 37], and the internal organs were collected for relative weight and histopathological evaluation.

### 2.5. Subchronic Toxicity Study

The study of subchronic oral toxicity was performed in accordance with the OECD guideline No. 408 [38] with a slight modification following the guidelines for preclinical and clinical trials of oriental medicine and herbal medicine outlined in the Decision of the Department of Science, Technology, and Training, Ministry of Health, Vietnam on [32]. The details were as follows: healthy adult male Swiss albino mice (23 ± 2 g, 6 weeks old) were divided into 5 groups (*n* = 6). Group 1 was the control group, and the other four groups were the test groups taking EtPV with doses of 100, 300, 500, and 700 mg/kg body weight (the formula to calculate the dose provided in the acute toxicity test) for 90 days. The mice were administered EtPV using the gastric feeding tube. Before being treated with EtPV, the mice were fasted overnight. Their weights were measured, while the blood and urine were collected for analysis of hematological parameters, serum biochemistry, and urine composition. During the observation of 14 days, the body weight was measured every week, while food intake and water intake were recorded every day [39].

On the 90^th^ day (end of treatment period), blood samples were taken for hematological and biochemical tests, and urine samples were also collected for urinalysis. The mice were sacrificed by inhaling CO_2_ at the end of the test. Liver, kidney, and heart were collected for histopathology [36, 37].

### 2.6. Clinical Observations and Survival

Mice were observed daily during 14 days of acute toxicity study and 90 days of subchronic toxicity study. In order to make the observation objective, all the observations and data recordings were implemented blindly. Group division of animals was unknown to the research workers who recorded the data. Clinical signs were observed before and after dose administration, preferably at the same time each day. Clinical observations included motor activity; tremors; convulsions; changes in gait; posture; stereotyped movements (e.g., excessive grooming) or bizarre behavior (e.g., walking backward), eyes activities: watery eyes and dilatation of the eyelids; skin activities: pallor and cyanosis; and general signs: salivation, diarrhea, dehydration (Robichaud test), difficult breathing, runny nose, aggression, and fear. The data were then compared with the control or baseline conditions [40].

### 2.7. Body Weight

The bodyweights of animals were measured weekly during the entire testing process by the GS-SHINKO (Japan) electronic balance test. The weight gain was calculated by the below formula [41]:

(1) $$\begin{array}{c} \text{Weight gain }\left(\%\right)=\frac{\left(W_{f}-\;\;W_{i}\right)}{W_{i}}\times 100, \end{array}$$

with *W*_*f*_ as the final weight and *W*_*i*_ as the initial weight.

### 2.8. Relative Organ Weight

On the experiment day, the mice were sacrificed by inhalation of CO_2_. Vital organs such as the liver, kidney, and heart were separated, weighed on an electronic scale (M), and compared with the control group [42]. Relative organ weight (ROW) was calculated by the formula: [43].

(2) $$\begin{array}{c} \text{ROW }\left(\%\right)=\frac{\text{Absolute visceral weight }\left(\text{g}\right)\;}{\text{body weight on the day of surgery }\left(\text{g}\right)}\times 100. \end{array}$$

### 2.9. Food and Water Consumption

The amount of food intake and water used by animals daily was recorded. Food and water intake were measured before being fed to each group. At the end of the day, the remaining food and water were collected, weighed to extrapolate the amounts of food and water that had been consumed by the animals during that day. The measuring unit for feeding was g/day, while the water consumption was measured in ml/day [44].

### 2.10. Hematological and Biochemical Study

The mice were fasted overnight before the test, anesthetized with chloroform, and the blood was collected. By the method of collecting mouse eye blood, we can get more than 1 ml of blood/mouse. Therefore, the amount of collected blood/mouse is sufficient for hematological and biochemical tests. The samples for hematology were put in bottles containing anticoagulant, ethylene diamine tetra-acetic acid (EDTA). Hematological analysis was performed using an automated hematology analyzer (Mythic 22, Germany) to obtain parameters including erythrocyte (RBC), hemoglobin (HGB), platelet (PLT), leukocyte (WBC), monocyte, lymphocyte, and granulocytes. Blood for biochemical analysis was gently placed in plain bottles to avoid hemolysis of the blood cells. The serum biochemical parameters were estimated using a clinical chemistry analyzer (Selectra E, Vitalab, Spankeren, Netherlands) with aspartate aminotransferase (AST), alanine aminotransferase (ALT), alkaline phosphatase (ALP), total protein, glucose, triglycerides, urea, and creatinine [45] being examined.

### 2.11. Histopathology Study

The mice were sacrificed by inhaling CO_2_ to collect vital internal organs. The heart, liver, and kidney have been separated from the mice. Absolute and relative organ weights were calculated, respectively. A portion of each organ was kept frozen in a -80-degree refrigerator for backup, while the rest was immersed in formaldehyde (10%). They were then cleaned in xylene and embedded in paraffin wax (melting at 60°C). A microtome was used to cut the mass of tissue embedded in paraffin into sections (5 *μ*m thick). The sections were then dried for 24 h at 37°C, reduced paraffin with xylene, and hydrated in descending concentrations of alcohol. They were thereafter stained with Mayer's hematoxylin and eosin dyes, dried, and observed on a light microscope (×100) [46]. The histopathological analysis was done by a certified histopathogist working at Division of Anatomic Pathology, Military Hospital 175, 786 Nguyen Kiem Street, Go Vap District, Ho Chi Minh City, Vietnam.

### 2.12. Urinalysis

Mice were placed individually in sterilized glasses cages, with water available but no food. Urine was collected 0.3 ml over 16 hours. The amount of water consumed and the amount of urine secreted were then measured. The test parameters including volume of urine, glucose, pH, the specific gravity of urine, ketones, blood cells, and protein in urine were analyzed by the Siemens Clinitek urine analyzer (Germany) [46].

### 2.13. Statistical Analysis

Results were presented as mean ± standard deviation (SD). The differences among groups were determined by one-way analysis of variance ANOVA followed Fisher's least significant difference using the Stagraphics Centurion XVI software (Statpoint Technologies Inc., Warrenton, Virginia, USA), and the criterion of statistical significance was set as *p* < 0.05.

## 3. Results and Discussion

### 3.1. Phytochemical Screening

The preliminary phytochemical screening of the extracts revealed the presence of alkaloids, flavonoids, saponins, sterol and triterpenoid, phenolic, tannins, phytosterol, diterpene, glycosides, protein, amino acid, and carbohydrate, and the absence of oils and fats in the extracts. The presence of the above substances in the ethanol extract of *Plukenetia volubilis* L. leaves agrees with the results of the research carried out by the Ana-Karina group in 2013 [10].

### 3.2. Behavioral Responses and General Appearance

**Figure 2:**
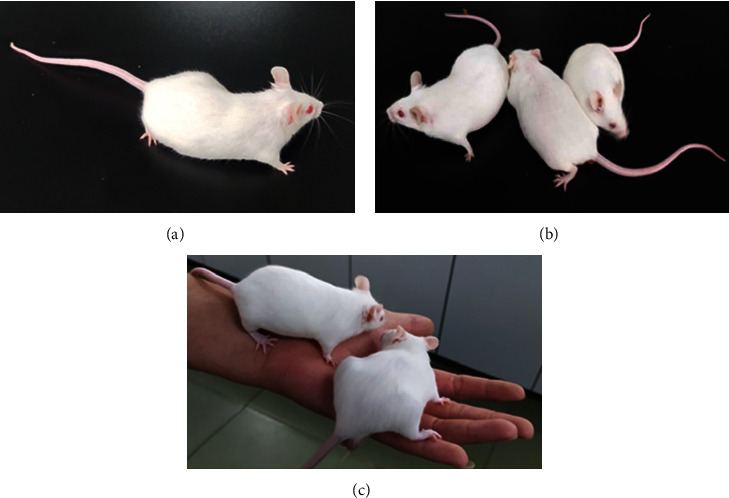
Mice in the EtPV toxicity test. (a) Mice in the control group. (b) Mice were orally administered with 700 mg/kg EtPV. (c) Mice were orally administered with 7000 mg/kg EtPV.

All animals survived the period of EtPV administration (Figure 2). There was no treatment-related mortality in any groups. Some signs such as a little restlessness, slow movement, and mild tremors appear to be the manifestation of the mouse's behavior. It can be concluded that oral doses up to 7000 mg/kg (acute) and 700 mg/kg (sub-chronic) of EtPV produce no abnormal behavior reactions and do not affect general appearance, as well as show no abnormalities or any symptoms of poisoning or death in mice (Figures 2(b) and 2(c)). A study carried out by the Gorriti group (2010) evaluating the toxicity of *P. volubilis* seed oil showed no signs of toxicity or death in mice and no change in behavioral responses and general appearance [47]. These results were similar to our results with respect to the behavioral responses of mice.

### 3.3. Body Weight, Food Intake, and Water Consumption

The food intake and water consumption of the five groups are presented in Table 1. In particular, average food intakes are 6.16 ± 1.28 g/day for the treatment group and 4.07 ± 1.62 g/day for the control group, while average water intakes are 6.71 ± 1.32 ml/day for the treatment group and 4.52 ± 1.24 ml/day for the control group, respectively. Although the food and water intakes of the tested groups increase, they remain within the ranges of food and water consumed by normal Swiss albino mice (food intake: 3.1 ± 0.1 to 6.3 ± 0.3 g/day; water intake: 3.9 ± 0.2 to 8.2 ± 0.3 ml/day) according to the conclusions from Abdulrahman (2004) [48]. The above results indicate that the EtPV does not induce appetite suppression and deleterious effects on the growth of mice. Normal health of mice suggests that there are no significant changes in their physiological as well as metabolic activity [49].

Significant changes in body weight and weight gains are detected in all treatment groups compared to the control group (Table 1). In particular, the weight gains of the treatment groups are 11% and 65% compared with 11% and 52% of the control groups in two toxicity assays. The increase of body weights of the mice treated with the EtPV may be due to the effect of saponins in the extract. They were found to be converted to sapogenin aglycon [50] which stimulated the nurturing centers in the mouse brain, consequently affecting food consumption level [51]. It was also reported in a relevant study performed by Rodeiro et al. (2018) examining the acute toxicity of the powder obtained from *P. volubilis* seed that the administration of material did not affect considerately body weight gain, food, and water consumption of the studied mice. There were also no significant differences in the hematological and biochemical parameters, weight of organs, and anatomical morphology and histological features between the control and the treatment groups reported in this work [52].

### 3.4. Hematological and Biochemical Analysis

The effects of *P. volubilis* leaf extract on the hematological parameters were displayed in Table 2. Compared to the control groups, no significant differences in lymphocyte, monocyte, and granulocyte counts were observed in EtPV-treated groups. However, the differences in RBC, PLT, and WBC between the control groups and the treatment groups using high doses of EtPV are noticeable. The RBC for the groups administered with 7000 mg/kg EtPV is 8.24 ± 0.45 × 10^6^ cell/mm^3^, while it is 7.39 ± 0.29 × 10^6^ cell/mm^3^ for the control group. The PLT is 699.56 ± 29.78 × 10^3^ cell/mm^3^ for the 700 mg/kg EtPV-treated group compared with 602.02 ± 23.56 × 10^3^ cell/mm^3^ for the control group. Additionally, the 7000 mg/kg EtPV-treated group has a WBC of 3.46 ± 0.47 × 10^3^ cell/mm^3^, whereas it is 2.56 ± 0.34 × 10^3^ cell/mm^3^ for the control group. Even though the RBC, PLT, and WBC of the treatment groups are higher than those of the control groups which can be attributed to mild body reactions due to the use of EtPV and/or individual variation of mice [53], these values still stay within the ranges corresponding to normal Swiss albino mice [54].

Variations in the level of ALT, AST, and ALP were noticed among the study groups (Table 3). Plasma ALT and AST levels in the serum decreased due to the administration of *P. volubilis* leaf extract at some selected doses. ALT of the 7000 mg/kg EtPV-treated groups is 69.01 ± 2.13 U/L, which is lower than that of the control group (69.69 ± 2.25 U/L). The level of AST of the 700 mg/kg EtPV-treated group is 98.02 ± 1.43 U/L, while the corresponding figure for the control group is 99.41 ± 1.84 U/L. The liver is considered as the body's shield preventing toxins entering the body through the digestive tract and at the same time reducing toxicity and eliminating wastes due to the body's metabolism. AST and ALT are indicators of liver damage, and when a large number of liver cells are damaged, AST and ALT will be “freed” and massively released into the bloodstream [50]. The plasma AST and ALT levels in the serum did not increase in mice taking EtPV indicating that the activity of the liver was preserved [55].

The kidney is responsible for excreting metabolic wastes and foreign chemicals, regulating the concentration of osmotic and electrolytes, which is evaluated via the expression of compositions of urea and creatinine [56]. Creatinine is a waste product produced by the daily movement of muscle tissue, while urea is the end product of protein metabolism. When creatinine and urea are produced, they are filtered by the kidneys and excreted in the urine, which is useful in measuring glomerular filtration. Significant reduction in plasma levels of urea and creatinine is found mostly in mice given EtPV. Specifically, the creatinine level for the 7000 mg/kg EtPV-treated group is 0.31 ± 0.57 mg/dl, lower than that of the control group at 0.25 ± 0.19 mg/dl. The urea level is 12.23 ± 2.09 mg/dl for the 700 mg/kg EtPV-treated group, while the value for the control group is 13.14 ± 2.04 mg/dl. Lower urea and creatinine parameters in the EtPV-treated group compared with the control group are an indication that the EtPV is not nephrotoxic.

The glucose levels and total protein in the EtPV-treated groups are higher than those of the control groups. In particular, the glucose level is 69.08 ± 3.05 mg/dl for the 700 mg/kg EtPV-treated group, whereas the value for the control group is 64.03 ± 2.22 mg/dl. The total protein of the group administered with 7000 mg/kg EtPV is 6.09 ± 0.12 g/dl compared with 5.59 ± 0.22 g/dl of the control group (*p* < 0.05). Meanwhile, the level of triglyceride in serum is 96.44 ± 2.04 mg/dl for the 7000 mg/kg EtPV group, lower than that of the control group (96.89 ± 2.11 mg/dl). They indicate no disturbances in the metabolism induced by EtPV intoxication. The hematological and biochemical parameters in both groups (control and treatment group) remain the ranges of hematology and biochemical parameters corresponding to normal Swiss albino mice [54].

### 3.5. Organ Weight

Unfavorable interactions between the plant extract with important organs in the body (heart, liver, and kidney) lead to cells being destroyed and inflamed, which are reflected in the organ/body ratio change (relative weight) and absolute weight of organs [55]. Visceral weight change is also a sensitive indicator showing the poisoning phenomenon in animals treated with extracts and, thus, manifest the toxicity of herbal extract [56]. The heart, liver, and kidney play important roles in metabolism and vital functions of these organs make them the subject of frequent attacks of toxic compounds [57]. The relative weight and absolute weight of the liver, kidney, and heart of the mice treated with EtPV show not much difference compared with the control group (Table 4). It therefore can be concluded that after using EtPV, the metabolic activity of the animal has not changed abnormally, and there is no manifestation of the body being attacked by harmful substances.

### 3.6. Anatomical Morphology and Histological Features

**Figure 3:**
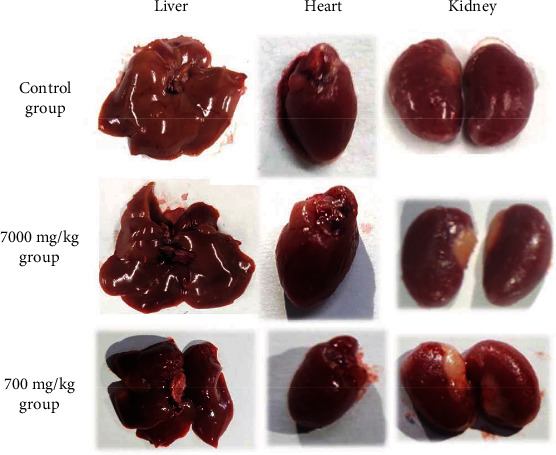
Photos of heart, liver, and kidneys from the acute and subchronic toxicity studies.

Macro examination of all internal organs including the stomach, small intestine, large intestine, spleen, lung, liver, heart, kidney, and testicular found no significant differences in location, shape, size, or overall color and texture of these organs between the groups. Gross morphology of the heart, liver, and kidney is displayed in Figure 3. The heart has a red, solid, 3-sided pyramid, very clear atrioventricular furrow that separates the atrium and ventricle. The liver has a triangular shape consisting of a protruding diaphragm surface, a flat visceral surface, and a circumference around the visceral surface which is the lower margin. The diaphragm surface of the liver has a smooth dome, close to the diaphragm, and the visceral surface has two longitudinal grooves and a horizontal H-shaped groove dividing the lower surface of the liver into lobes. The kidneys are reddish-brown, smooth on the surface due to being wrapped in a fibrous, pea-shaped bag.

**Figure 4:**
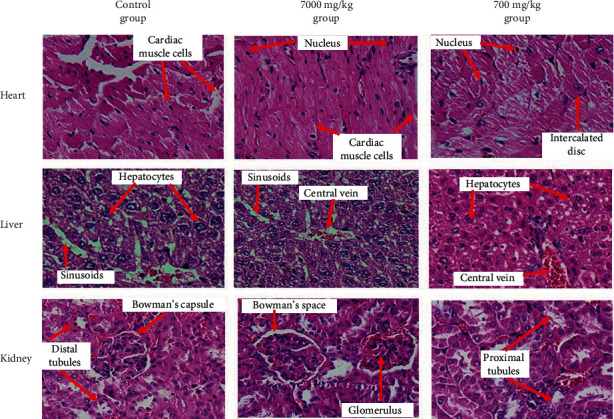
Photomicrographs (magnification 100) of the sections of the heart, liver, and kidney showing normal features (red arrow symbol) in mice treated orally EtPV at the models' acute and subchronic toxicity. The sections were stained with Hematoxylin (H) và Eosin (E).

Histological studies showed no abnormalities in the liver, kidney, and heart tissue in the EtPV-treated mice compared to the control group (Figure 4). Histology of the liver sections of the EtPV-treated animals showed normal hepatic cells with well-preserved cytoplasm, prominent nucleus, nucleolus, and visible central veins, and blood vessels have normal structure. Kidney tissue micrographs of the extract-administered groups indicated no significant changes in kidney cells as evidenced by the absence of tubular degeneration and desquamation, tubular necrosis, mononuclear cell infiltration, and intertubular hemorrhage compared to the normal control group. Cardiac tissue has an endocardium with an endothelial layer and a thin subendothelium containing collagen fibers, elastic fibers, and fibroblasts. The heart muscles are connected in a mesh that runs in a spiral direction. The results of histopathological studies indicated that the EtPV does not have any adverse effects on the morphology of tissues, and these observations support the above biochemical results.

### 3.7. Urinalysis

In clinical studies, the determination of the composition of urine is of great meaning in diagnosis and revealing substance metabolism. Changes in urine parameters reflect metabolic disorders of the body [58]. In the present toxicity tests, there are no differences in specific gravity, pH, and urine volume between the EtPV and control groups (Table 5). For example, urine volume is 16.5 ± 0.6 ml for the 7000 mg/kg EtPV-treated group compared with 16.7 ± 0.5 ml for the control group. The specific gravity is 1.013 ± 0.004 for the 700 mg/kg EtPV group, while the figure for the control group is 1.011 ± 0.004. There are no blood cells in the urines and the tests for glucose, and protein and ketone were detected indicating the absence of these substances in all groups. Therefore, it can be concluded that the EtPV is not toxic to the kidney at any of the tested doses [59].

## 4. Conclusions

Toxicity study of the ethanol extract of *P. volubilis* L. leaves revealed that they did not produce any adverse effects on overall mortality and behavior in Swiss albino mice at tested doses. Therefore, the oral LD_50_ of EtPV is greater than 7000 mg/kg. The results of acute and subchronic toxicity survey showed that EtPV does not adversely affect body weight, histopathology of the heart, liver, and kidney; hematological parameters; blood biochemistry; and urine parameters. No sign of toxicity was observed in the treated mice. The results, therefore, have shown a clear picture of the safety of EtPV. Overall, the EtPV is safe to consume in the concentration range up to 7000 mg/kg.

## Figures and Tables

**Table 1:** The effect of EtPV on the food intake, water consumption, and body weight of mice by acute and subchronic toxicity assays.

Group	Average food intake (g/day)	Average water intake (ml/day)	Body weight	
			Actual weight (g)	Weight gain (%)
Acute oral toxicity				
Control group	4.07 ± 1.62	4.74 ± 0.62	24.21 ± 0.1	0.11 ± 0.01
1000 mg/kg	5.63 ± 1.91	5.56 ± 1.34	24.36 ± 0.59	0.12 ± 0.03
3000 mg/kg	4.67 ± 1.37	5.78 ± 1.12	25.17 ± 0.12^∗^	0.13 ± 0.02
5000 mg/kg	5.98 ± 1.52	5.45 ± 0.98	24.56 ± 0.48	0.12 ± 0.01
7000 mg/kg	6.16 ± 1.28	6.08 ± 0.51^∗^	25.03 ± 0.67	0.11 ± 0.03
Subchronic oral toxicity				
Control group	4.24 ± 1.24	4.52 ± 1.24	26.72 ± 0.84	0.52 ± 0.03
100 mg/kg	4.99 ± 1.13	5.74 ± 1.13	27.15 ± 0.51	0.57 ± 0.02
300 mg/kg	6.01 ± 1.32	6.39 ± 1.31	27.79 ± 0.72	0.61 ± 0.01
500 mg/kg	5.61 ± 1.22	5.08 ± 1.13	28.02 ± 0.63	0.69 ± 0.02
700 mg/kg	6.25 ± 1.11	6.71 ± 1.32	27.48 ± 0.54	0.65 ± 0.02

Values are displayed as means ± SD. Value that is significantly different from the corresponding value of the control group (^∗^*p* < 0.05) was marked with an asterisk.

**Table 2:** The effect of EtPV on the hematological parameters in mice by acute and subchronic toxicity assays.

Group	RBC (×10^6^cell/mm^3^)	HGB (g/dl)	PLT (×10^3^cell/mm^3^)	WBC (×10^3^cell/mm^3^)	Lymphocyte (%)	Monocyte (%)	Granulocytes (%)
Acute oral toxicity							
Control group	7.39 ± 0.29	11.33 ± 0.44	575.61 ± 54.26	2.56 ± 0.34	39.76 ± 2.14	5.69 ± 0.21	54.55 ± 2.21
1000 mg/kg	7.81 ± 0.57	11.47 ± 0.51	628.68 ± 42.57	2.79 ± 0.29	41.45 ± 1.34	5.93 ± 0.56	52.62 ± 1.46
3000 mg/kg	7.97 ± 0.78	12.39 ± 0.37	679.82 ± 38.66	3.05 ± 0.53	40.66 ± 1.56	5.98 ± 0.48	53.36 ± 2.13
5000 mg/kg	8.27 ± 0.12^∗^	11.88 ± 0.66	652.36 ± 47.13	3.28 ± 0.41	41.45 ± 1.72	6.07 ± 0.65	52.48 ± 1.45
7000 mg/kg	8.24 ± 0.45	12.46 ± 0.59	698.47 ± 61.72	3.46 ± 0.47	41.99 ± 2.48	6.18 ± 0.17^∗^	51.83 ± 1.58
Subchronic oral toxicity							
Control group	7.57 ± 0.19	12.51 ± 0.33	602.02 ± 23.56	2.56 ± 0.25	39.46 ± 1.21	5.24 ± 0.15	53.28 ± 1.21
100 mg/kg	8.03 ± 0.42	12.68 ± 0.24	624.56 ± 35.43	2.89 ± 0.24	40.87 ± 1.16	5.36 ± 0.19	54.54 ± 1.25
300 mg/kg	7.99 ± 0.55	13.01 ± 0.28	648.68 ± 41.11	2.75 ± 0.19	39.95 ± 1.22	5.94 ± 0.36	54.02 ± 1.32
500 mg/kg	8.18 ± 0.48	12.89 ± 0.16	687.61 ± 39.45	3.01 ± 0.28	41.45 ± 1.80	5.56 ± 0.17	53.98 ± 1.19
700 mg/kg	8.84 ± 0.17^∗^	13.12 ± 0.23	699.56 ± 29.78^∗^	2.99 ± 0.26	42.09 ± 1.26	5.64 ± 0.43	54.77 ± 1.37

Values are displayed as means ± SD. Value that is significantly different from the corresponding value of the control group (^∗^*p* < 0.05) was marked with an asterisk.

**Table 3:** The effect of EtPV on the serum biochemical parameters in mice by acute and sub-chronic toxicity assays.

Group	Total proteins (g/dl)	Glucose (mg/dl)	Trygliceride (mg/dl)	ALT (U/L)	AST (U/L)	ALP (U/L)	Creatinine (mg/dl)	Urea (mg/dl)
Acute oral toxicity								
Control group	5.59 ± 0.22	62.77 ± 1.93	96.89 ± 2.11	69.69 ± 2.25	97.77 ± 1.26	130.89 ± 3.79	0.25 ± 0.19	12.59 ± 1.23
1000 mg/kg	5.99 ± 0.46	63.54 ± 2.76	97.43 ± 1.88	71.28 ± 2.16	98.46 ± 2.23	130.55 ± 4.64	0.43 ± 0.31	14.76 ± 1.39
3000 mg/kg	5.74 ± 0.57	64.97 ± 3.07	99.39 ± 2.22	70.98 ± 1.22	99.74 ± 1.38	134.36 ± 6.12	0.49 ± 0.46	14.03 ±2.16
5000 mg/kg	5.85 ± 0.79	64.46 ± 1.78	98.75 ± 1.27	70.32 ± 2.08	98.98 ± 2.31	133.28 ± 5.22	0.38 ± 0.11^∗^	13.37 ± 2.34
7000 mg/kg	6.09 ± 0.12^∗^	65.58 ± 2.33	96.44 ± 2.04	69.01 ± 2.13	97.47 ± 1.44	129.03 ± 3.26	0.31 ± 0.57	12.18 ± 2.28
Subchronic oral toxicity								
Control group	6.21 ± 0.16	64.03 ± 2.22	97.76 ± 1.75	70.67 ± 2.04	99.41 ± 1.84	128.56 ± 4.67	0.51 ± 0.13	13.14 ± 2.04
100 mg/kg	6.44 ± 0.22	62.77 ± 3.09	99.22 ± 2.24	71.56 ± 1.19	98.89 ± 1.91	134.79 ± 5.04	0.65 ± 0.12	13.95 ± 1.67
300 mg/kg	6.51 ± 0.29	67.19 ± 3.06	100.34 ± 1.56	72.17 ± 2.02	101.55 ± 1.72	133.54 ± 5.02	0.47 ± 0.16	15.07 ± 2.08
500 mg/kg	6.53 ± 0.18^∗^	68.14 ± 2.57	98.55 ± 2.18	71.98 ± 1.16	100.36 ± 1.84	131.29 ± 5.08	0.61 ± 0.11	14.62 ± 1.34
700 mg/kg	6.72 ± 0.24	69.08 ± 3.05	96.42 ± 1.45	69.24 ± 1.26	98.02 ± 1.43	125.11 ± 4.71	0.31 ± 0.19	12.23 ± 2.09

Values are displayed as means ± SD. Value that is significantly different from the corresponding value of the control group (^∗^*p* < 0.05) was marked with an asterisk.

**Table 4:** The effect of EtPV on the relative organ weight of the heart, liver, and kidney in mice measured by acute and subchronic toxicity assays.

Group	Heart weight		Liver weight		Kidney weight	
	M (g)	ROW (%)	M (g)	ROW (%)	M (g)	ROW (%)
Acute oral toxicity						
Control group	0.18 ± 0.02	0.69 ± 0.02	1.13 ± 0.29	4.64 ± 0.19	0.25 ± 0.19	1.02 ± 0.14
1000 mg/kg	0.17 ± 0.01	0.73 ± 0.01	1.13 ± 0.24	4.51 ± 0.22	0.24 ± 0.22	1.01 ± 0.12
3000 mg/kg	0.18 ± 0.01	0.75 ± 0.03	1.15 ± 0.31	4.69 ± 0.31	0.24 ± 0.18	1.02 ± 0.08
5000 mg/kg	0.17 ± 0.03	0.69 ± 0.01	1.14 ± 0.26	4.35 ± 0.26	0.25 ± 0.36	1.08 ± 0.11
7000 mg/kg	0.18 ± 0.02	0.74 ± 0.02	1.14 ± 0.33	4.42 ± 0.34	0.23 ± 0.34	1.03 ± 0.07
Subchronic oral toxicity						
Control group	0.22 ± 0.02	0.82 ± 0.03	1.28 ± 0.12	4.79 ± 0.23	0.29 ± 0.03	1.09 ± 0.09
100 mg/kg	0.23 ± 0.01	0.85 ± 0.02	1.37 ± 0.14	5.05 ± 0.28	0.31 ± 0.02	1.14 ± 0.07
300 mg/kg	0.24 ± 0.03	0.86 ± 0.03	1.33 ± 0.11	4.79 ± 0.36	0.31 ± 0.03	1.16 ± 0.08
500 mg/kg	0.24 ± 0.02	0.86 ± 0.04	1.38 ± 0.13	4.93 ± 0.24	0.32 ± 0.02	1.15 ± 0.05^∗^
700 mg/kg	0.23 ± 0.01	0.84 ± 0.03	1.33 ± 0.14	4.81 ± 0.29	0.31 ± 0.01	1.13 ± 0.06

Values are displayed as means ± SD. Value that is significantly different from the corresponding value of the control group (^∗^*p* < 0.05) was marked with an asterisk.

**Table 5:** The effect of EtPV on the urinalysis parameters by mice of acute and sub-chronic toxicity.

Group	Volume (ml)	Specificity gravity	pH	Glucose (mg/dl)	Protein (mg/dl)	Ketones (mg/dl)	Blood cell (cells/*μ*l)
Acute oral toxicity							
Control group	16.7 ± 0.5	1.012 ± 0.001	7.0 ± 0.1	Nil	Nil	Nil	Nil
1000 mg/kg	17.6 ± 0.1^∗^	1.011 ± 0.002	7.0 ± 0.1	Nil	Nil	Nil	Nil
3000 mg/kg	16.9 ± 0.7	1.012 ± 0.002	7.0 ± 0.2	Nil	Nil	Nil	Nil
5000 mg/kg	16.7 ± 0.5	1.012 ± 0.001	7.0 ± 0.2	Nil	Nil	Nil	Nil
7000 mg/kg	16.5 ± 0.6	1.011 ± 0.001	6.9 ± 0.2	Nil	Nil	Nil	Nil
Subchronic oral toxicity							
Control group	16.9 ± 0.3	1.011 ± 0.004	6.9 ± 0.1	Nil	Nil	Nil	Nil
100 mg/kg	16.3 ± 0.4	1.011 ± 0.002	6.9 ± 0.2	Nil	Nil	Nil	Nil
300 mg/kg	17.1 ± 0.7	1.012 ± 0.003	6.8 ± 0.1	Nil	Nil	Nil	Nil
500 mg/kg	17.5 ± 0.6	1.014 ± 0.005	7.0 ± 0.2	Nil	Nil	Nil	Nil
700 mg/kg	16.7 ± 0.4	1.013 ± 0.004	6.9 ± 0.2	Nil	Nil	Nil	Nil

Values are displayed as means ± SD. Value that is significantly different from the corresponding value of the control group (^∗^*p* < 0.05) was marked with an asterisk.

## Data Availability

The data in form of tables and figures used to support the findings of this study are included within the article.
